# Supplementary material for: α-Synuclein Overexpression and the Microbiome Shape the Gut and Brain Metabolome in Mice
Source: bioRxiv. 2024 Jun 10:2024.06.07.597975. Preprint. [Version 1] doi: 10.1101/2024.06.07.597975 (PMC11195096; doi:10.1101/2024.06.07.597975)
Supplement: Supplement 2 [file NIHPP2024.06.07.597975v1-supplement-2.pdf]

bioRxiv preprint doi: https://doi.org/10.1101/2024.06.07.597975; this version posted June 10, 2024. The copyright holder for this preprint (which was not certified by peer review) is the author/funder, who has granted bioRxiv a license to display the preprint in perpetuity. It is made available under aCC-BY 4.0 International license.

Supplementary Figures

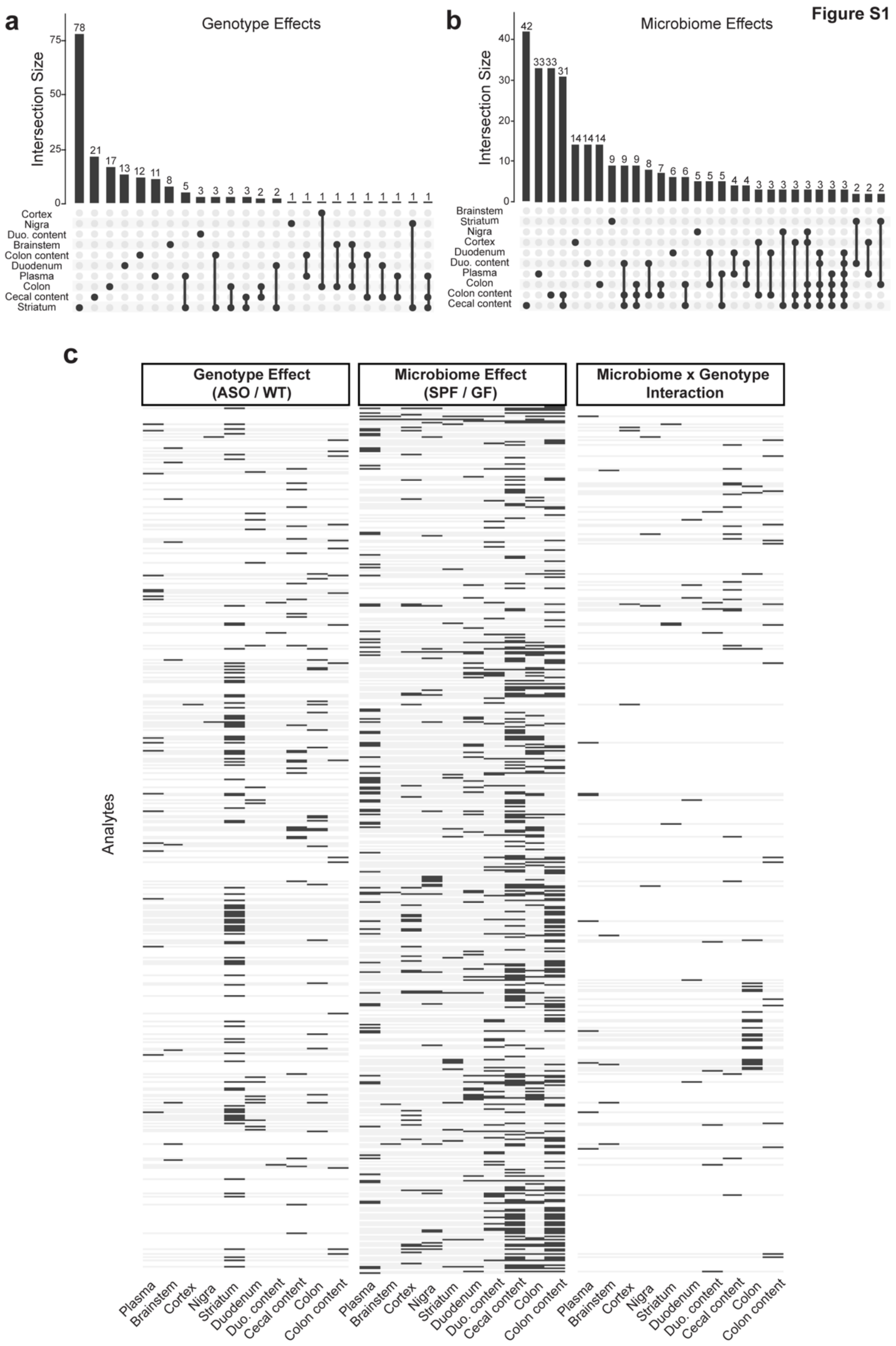

bioRxiv preprint doi: https://doi.org/10.1101/2024.06.07.597975; this version posted June 10, 2024. The copyright holder for this preprint (which was not certified by peer review) is the author/funder, who has granted bioRxiv a license to display the preprint in perpetuity. It is made available under aCC-BY 4.0 International license.

Fig. S1: a-b) UpSet plots of unique and shared metabolite sets across all samples. Interaction size describes the number of metabolites with a significant genotype (**a**) or microbiome (**b**) interaction effect ($p<0.05$). The dots below the bar chart indicate the tissue source of the metabolites. Singular points with no vertical lines connecting to other tissues indicate a set of metabolites which are uniquely altered in a particular tissue. **c**) Binary heatmap of metabolites significantly altered in at least one tissue for at least one variable (genotype, microbiome, microbiome×genotype) from the linear model.

bioRxiv preprint doi: https://doi.org/10.1101/2024.06.07.597975; this version posted June 10, 2024. The copyright holder for this preprint (which was not certified by peer review) is the author/funder, who has granted bioRxiv a license to display the preprint in perpetuity. It is made available under aCC-BY 4.0 International license.

Figure S2

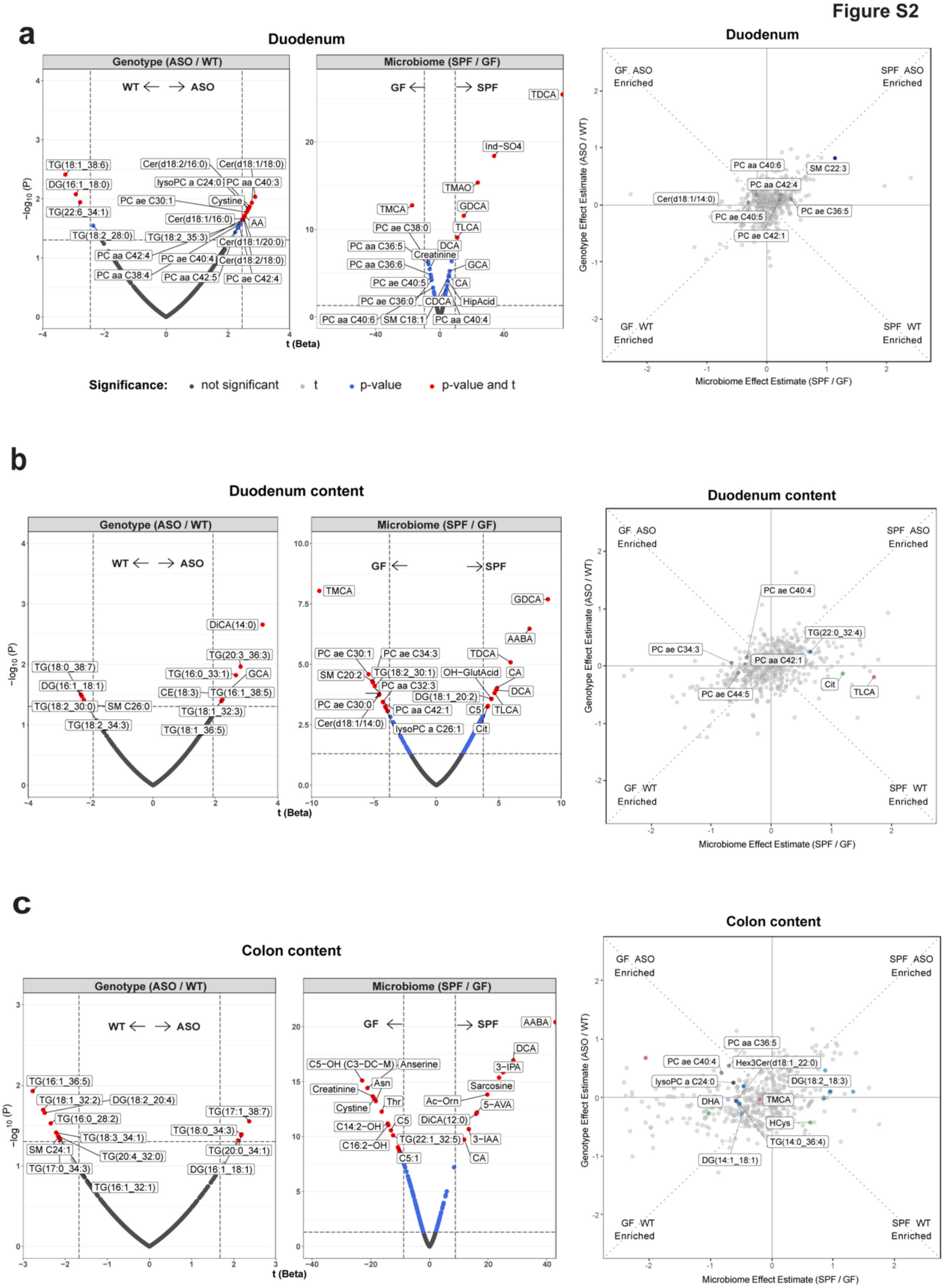

Fig. S2: Volcano plots and scatterplots of the most significantly altered metabolites showing genotype, microbiome, or genotype×microbiome interaction effects in the duodenum (**a**), duodenal contents (**b**), or colonic contents (**c**). Conventions are as in Fig. 2.

bioRxiv preprint doi: https://doi.org/10.1101/2024.06.07.597975; this version posted June 10, 2024. The copyright holder for this preprint (which was not certified by peer review) is the author/funder, who has granted bioRxiv a license to display the preprint in perpetuity. It is made available under aCC-BY 4.0 International license.

Figure S3

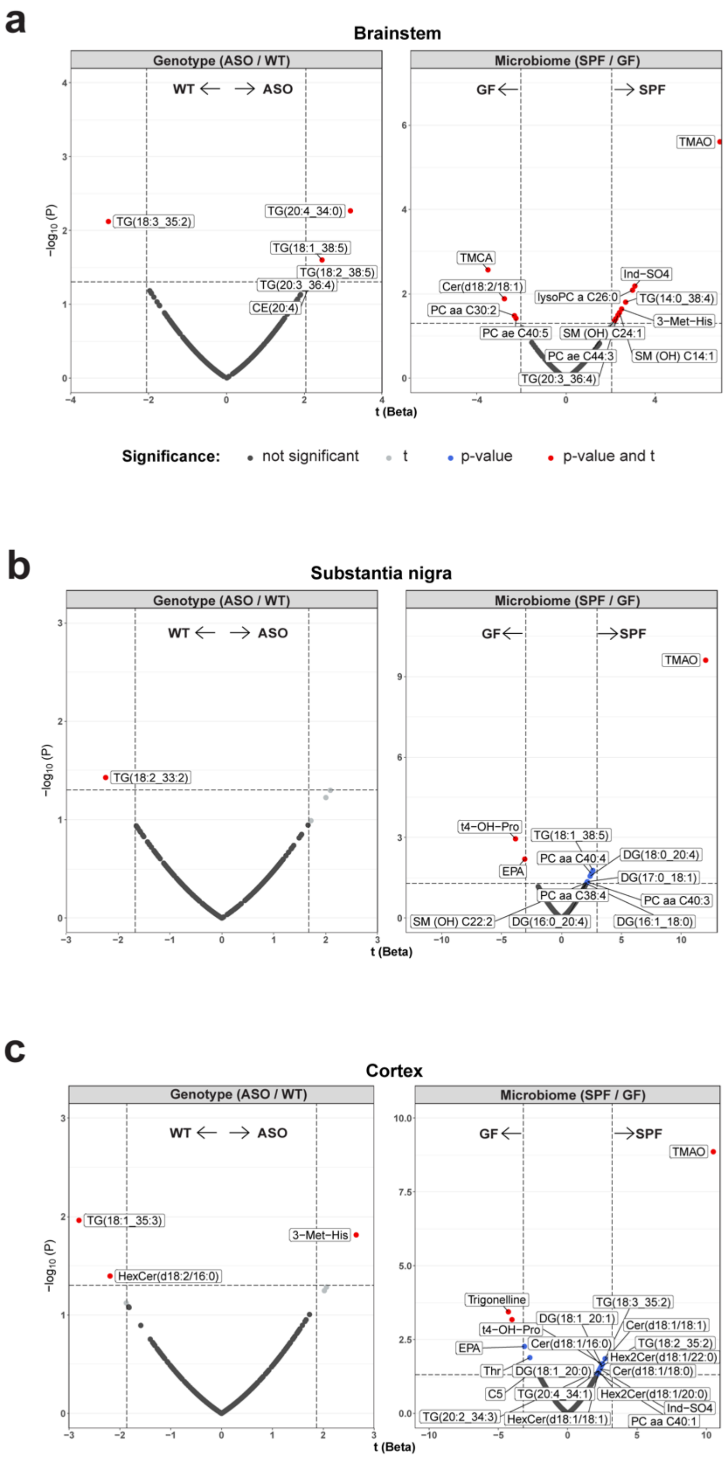

Fig. S3: Volcano plots of metabolites showing genotype or microbiome effects in the brainstem (**a**), substantia nigra (**b**) and cortex (**c**).
